# Factors associated with caregiver strain among mothers and fathers of children with advanced cancer

**DOI:** 10.1017/S1478951524001196

**Published:** 2024-10-04

**Authors:** Anna L. Olsavsky, Malcolm Sutherland-Foggio, Charis J. Stanek, Kylie N. Hill, Alexandra C. Himelhoch, Ansley E. Kenney, Lisa Humphrey, Randal Olshefski, Micah A. Skeens, Leena Nahata, Cynthia A. Gerhardt

**Affiliations:** 1 The Center for Biobehavioral Health at the Abigail Wexner Research Institute at Nationwide Children’s Hospital, Columbus, OH, USA;; 2 Department of Clinical and Health Psychology, University of Florida, Columbus, FL, USA;; 3 Department of Psychology, University of Wisconsin-Milwaukee, Milwaukee, WI, USA;; 4 Nationwide Children’s Hospital, Columbus, OH, USA; 5 College of Medicine, The Ohio State University, Columbus, OH, USA

**Keywords:** Advanced pediatric cancer, caregiver strain, family roles, fathers, stress

## Abstract

**Objectives.:**

To characterize caregiver experiences in the context of advanced pediatric cancer by identifying individual (i.e., demographic factors, stress) and family context factors (i.e., family roles, social support) associated with caregiver strain.

**Methods.:**

Families of children (ages 5–25) with advanced cancer (i.e., physician-estimated prognosis < 60%, relapsed/refractory disease) were recruited from a large children’s hospital. Mothers (*n* = 55; 87% White) and fathers (*n* = 30; 83% White) reported on their caregiver strain, cancer-specific stress, general stress, social support, division of 7 family roles (e.g., medical care of ill child, household chores), and their satisfaction with each role.

**Results.:**

Parents reported moderate caregiver strain, cancer-specific stress, and general stress, and high social support and satisfaction with family roles. Fathers reported family roles were shared equally, whereas mothers reported either sharing roles or completing them independently. When accounting for income and partnership status, greater caregiver strain for mothers was associated with greater general stress, greater satisfaction with family roles, and lower social support. For fathers, greater caregiver strain was associated only with greater cancer-specific stress.

**Significance of results.:**

In the context of advanced pediatric cancer, fathers may experience caregiver strain as cancer-specific stress increases, whereas mothers’ strain may depend on broader family and social factors. Psychosocial providers should address general and cancer-specific stress within families, and provide resources for enhancing mothers’ social support. Additional research is needed with larger, more diverse samples to inform future intervention approaches.

## Introduction

Each year, over 15,000 U.S. children are diagnosed with cancer, which remains the leading cause of death by disease for children (Siegel et al. 2023). These families must accommodate demanding treatment schedules while managing emotional well-being during a difficult and uncertain time (Long and Marsland 2011; Granek et al. 2014; Wakefield et al. 2014; Molinaro and Fletcher 2018; Peikert et al. 2020; Hjelmstedt et al. 2021). Advanced disease can result in more intensive therapy and greater psychological distress (Rosenberg et al. 2013). Thus, parents may be at-risk for caregiver strain due to the emotional burden and need to balance demands from work, home, and hospital (Pai et al. 2007; Ozdemir Koyu and Tas Arslan 2021; Chaghazardi et al. 2022).

Caregiver strain is defined as difficulties, burdens, and negative effects caregivers experience due to caring for a loved one with a health-related condition (Brannan et al. 1997; Brannan and Heflinger 2001). According to Brannan’s double ABCX model of caregiver strain and psychological distress, caregiver strain is the result of child factors (e.g., medical symptoms), life stressors, and family context (e.g., resources, perceptions) (Brannan and Heflinger 2001). In the context of pediatric cancer, there are established associations between greater caregiver strain, more severe disease (Salvador et al. 2015; Edmond et al. 2016; Boztepe et al. 2019; Ozdemir Koyu and Tas Arslan 2021), and lower levels of parental social support (Ozdemir Koyu and Tas Arslan 2021). However, compared to child factors (Salvador et al. 2015; Edmond et al. 2016; Boztepe et al. 2019; Ozdemir Koyu and Tas Arslan 2021), associations between family context and caregiver strain have received less attention and are predominantly informed by maternal perspectives (Pai et al. 2007; Sultan et al. 2016). Additionally, the contribution of family roles to caregiver strain is less known (Demirtepe-Saygılı and Bozo 2011; Hjelmstedt et al. 2021).

In the context of pediatric cancer, mothers are often primary caregivers, while fathers balance work and home responsibilities (Clarke et al. 2009; Jones et al. 2010; Hjelmstedt et al. 2021). Given both roles may be challenging (Quittner et al. 1998; Jones et al. 2010; Demirtepe-Saygılı and Bozo 2011), understanding their contribution to caregiver strain can inform tailored support for parents. However, limited research has examined fathers, paternal perspectives, and the division of caregiving (Brannan and Heflinger 2001; Jones et al. 2010; Demirtepe-Saygılı and Bozo 2011; Hjelmstedt et al. 2021; Ozdemir Koyu and Tas Arslan 2021). Although knowledge is limited regarding how unmet parental needs affect the ill child and family system (Brannan et al. 1997; Kearney et al. 2015; Brennan et al. 2022), it is known that parent and child distress are often linked in pediatric cancer (Bakula et al. 2019). Additionally, research within other pediatric illness groups indicates reductions in caregiver strain are associated with improvements in child psychological outcomes (Accurso et al. 2015; Schleider et al. 2015; Brennan et al. 2022). Thus, to optimally support the well-being of families affected by advanced pediatric cancer, it is critical to understand maternal and paternal perspectives of the family context and examine how family and individual factors might influence parents’ caregiver strain. Therefore, with a goal of examining the understudied family context component of Brannan’s ABCX model (Brannan and Heflinger 2001), our aim was to identify individual factors (i.e., demographic factors, stress) and family factors (i.e., family roles, social support) associated with caregiver strain in the context of advanced pediatric cancer. We expected that mothers and fathers with higher role satisfaction, greater social support, and lower general and cancer-specific stress would experience less caregiver strain. Given the lack of representation of fathers’ perspectives in existing literature (Pai et al. 2007; Sultan et al. 2016), we also explored contrasting paternal and maternal perspectives of family and caregiving experiences.

## Methods

### Procedure

Data were from a larger, IRB-approved (IRB16–00869) study examining symptom burden, quality of life, and family goals for care among children with advanced pediatric cancer and their parents. Families were identified through oncology or palliative care teams and hospital census at a large children’s hospital from 2017 to 2022. After consent/assent, families chose to complete surveys online or on paper.

### Participants

Families were eligible if their child with advanced cancer was 5–25 years of age, spoke English, had at least one parent (≥ 18 years of age) who spoke English, and lived within 140 miles of the hospital. Advanced cancer was defined as physician-estimated prognosis for survival less than 60%, relapsed or refractory disease, or referral to end-of-life care. Children with significant developmental disabilities were excluded.

Of 149 families approached, 72 (48%) participated. Because children ≥ 18 years old could participate independently, a total of 55 mothers and 30 fathers representing 66 families had complete data and were included in this paper (Table 1). Three grandmothers, 1 aunt, 1 stepmother, and 1 uncle were included as “parents,” “mothers,” and “fathers” for analytical purposes given that they were all caregivers of their child with cancer. Of these 66 families, 35 had only the mother(s) enroll, 11 had only the father, and 20 families had both. Parents were primarily White (>80%), married (58.2% of mothers; 83.3% of fathers), non-Hispanic (>98%), of middle to upper income levels (>55% earning >$50,000 USD), biological parents (>90%) of the enrolled child, and had some college education (>75%; see Table 1). Of 20 paired parents, 17 were married, 2 were divorced, and 1 was separated and living with someone. Parents were asked to select 1 option to best describe their current partnership status: single, married, divorced, separated, remarried, widowed, or living with someone. For analyses, married or living with someone was considered “partnered,” whereas single, divorced, separated, or widowed was considered “single.”

The sample of children was on average 12 years old, male (59%), and 2.45 (*SD* = 3.05) years post-diagnosis (66.7% relapsed/refractory disease). Most children were diagnosed with a solid tumor (42.4%), and were receiving therapy rated highest in intensity (65.2%) based on the Intensity of Treatment Rating Scale (ITR-3) (Kazak et al. 2012).

### Measures

#### Caregiver strain

The 20-item Caregiver Strain Questionnaire (CSQ) assessed the extent of difficulties due to caregiver roles and responsibilities on a scale of 1 (“not at all”) to 5 (“very much”) (Brannan et al. 1997). It included 3 dimensions: objective strain (10 items; observable burden from the child’s diagnosis), subjective internalized strain (6 items; inwardly-directed negative feelings like worry, sadness, fatigue), and subjective externalized strain (4 items; child-directed negative feelings like anger or resentment). Overall caregiver strain scores were used in analyses and demonstrated good internal consistency (α_mothers_ = .90; α_fathers_ = .85).

#### Family roles questionnaire

**Figure 1.**
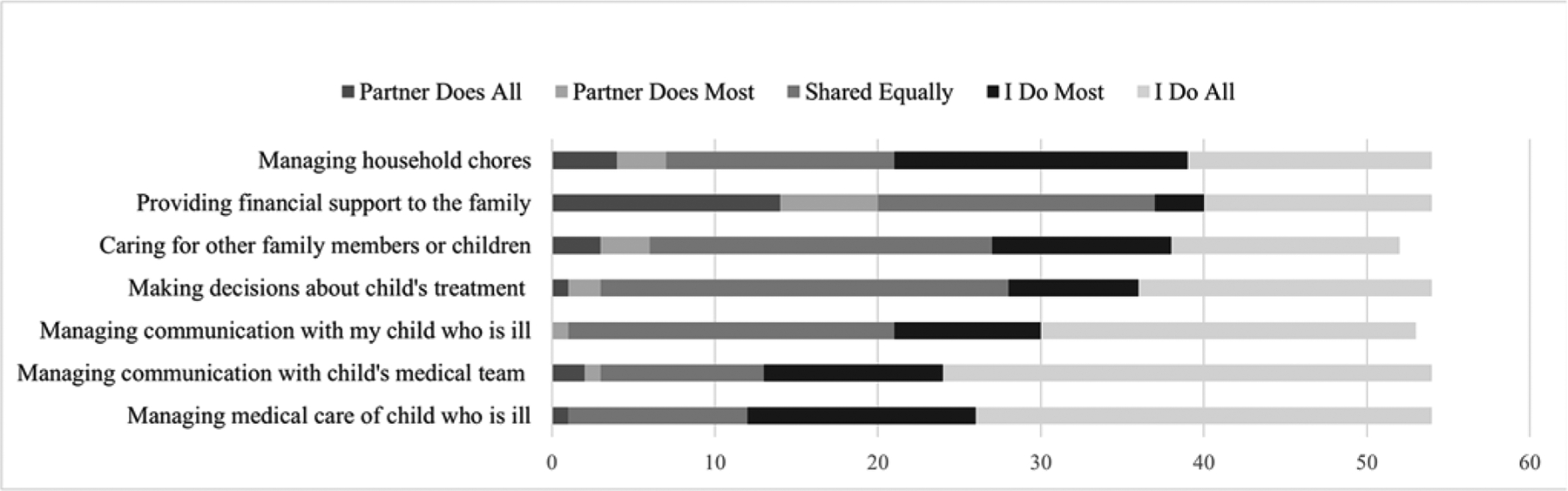
Mother’s reports of family roles.

**Figure 2.**
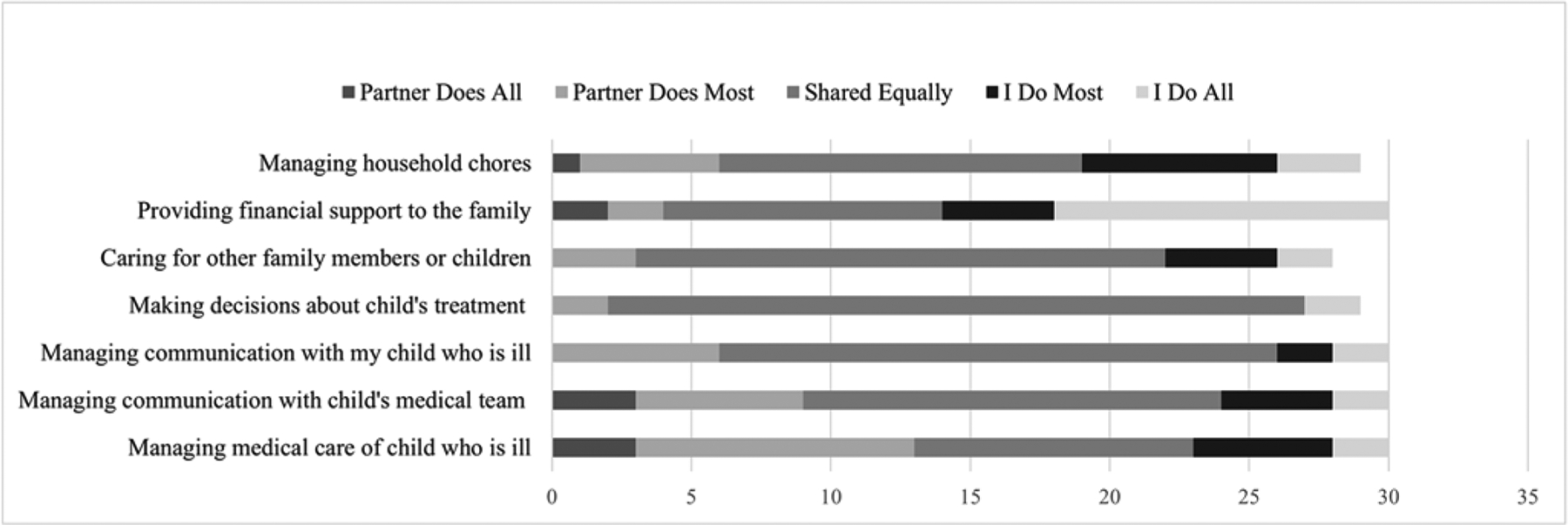
Father’s reports of family roles.

Using a measure adapted from previous work (Quittner et al. 1998), parents reported the frequency they completed 7 roles relative to their partner and their level of satisfaction with this arrangement. Four roles were hospital-related, whereas 3 were external (see Figures 1 and 2). Frequency of roles were reported as: 0 – My spouse or partner usually does all of it, 1 – My spouse or partner usually does most of it, 2 – We usually share this role equally, 3 – I usually do most of it, or 4 – I usually do all of it. Satisfaction was rated on a 0 to 3 scale (“not at all satisfied” to “very satisfied”). Internal consistency was acceptable for frequency (α_mothers_ = .84; α_fathers_ = .80) and satisfaction (α_mothers_ = .92; α_fathers_ = .90).

#### Cancer-specific stress

Mothers and fathers reported their cancer-specific stress using the Responses to Stress Questionnaire-Advanced Cancer (RSQ-AC) (Compas et al. 2006). Parents rated 12 items (e.g., not being able to help their child feel better, paying bills and family expenses, not knowing if the child’s illness will get better) from 1 to 4 (“not at all stressful” to “very stressful”). Internal consistency was good (α_mothers_ = .80; α_fathers_ = .85)

#### General stress

The Perceived Stress Scale (PSS) assessed mother and father general stress (Cohen et al. 1983). Parents rated 10 items from 0 (“never”) to 4 (“very often”) based on how often they experienced the stressors. The PSS demonstrated good internal consistency (α_mothers_ = .89; α_fathers_ = .86).

#### Social support

The Medical Outcomes Study Social Support Survey (MOS-SSS) (Sherbourne and Stewart 1991) assessed parents’ social support (i.e., emotional and informational support, tangible support, affectionate support, positive social interaction). Two items were added to assess financial and childcare assistance. Mothers and fathers rated items from 1 to 5 (“none of the time” to “all of the time”), indicating the degree to which they had each type of support. A mean score was calculated based on these 21 items. An additional global item assessed satisfaction with overall social support. The global item was highly correlated with the mean score, *r* = .87–.88, *p* < .001; therefore, the mean score was used in analyses. The mean score had excellent internal consistency (α_mothers_ = .97; α_fathers_ = .98).

#### Child medical characteristics

Medical chart data (e.g., diagnosis, prognosis) were abstracted, and the ITR-3 was used to classify treatment intensity from 1 to 4 (“least intensive” to “most intensive”) (Kazak et al. 2012).

### Analysis plan

Descriptive statistics were calculated for variables of interest (i.e., caregiver strain, family roles, cancer-specific stress, general stress, social support). Pearson correlations and *t*-tests explored associations and differences between paired mother–father dyads’ variables of interest. Differences between single and partnered mothers’ family role frequency were also explored using independent samples *t*-tests. Hierarchical regressions evaluated factors associated with caregiver strain for mothers and fathers. Step 1 included demographic characteristics (i.e., partnership status, income) for mothers, but were excluded for fathers due to the small sample. Step 2 included individual factors (i.e., cancer-specific stress, general stress). The final step included family factors, (i.e., family role frequency and satisfaction, social support). Assumptions of all analyses were examined and met. Analyses were completed in IBM SPSS Statistics version 28 software (Corp 2019). Given low levels of missingness, missing data were handled using listwise deletion.

## Results

### Descriptives and bivariate associations with caregiver strain

Mothers reported moderate caregiver strain (*M* = 2.88, *SD* = 0.67), general stress (*M* = 2.07, *SD* = 0.72), and cancer-specific stress (*M* = 2.76, *SD* = 0.57). However, they also reported high social support (*M* = 4.02, *SD* = 0.93) and satisfaction with family roles (*M* = 2.45, *SD* = 0.70). Mothers most frequently reported doing all hospital-related roles (i.e., day-to-day child medical care, communication with medical team and child) and sharing responsibilities with partners in medical decision making for their ill child, caring for other family or children at home, and providing financial support (Figure 1). Independent samples *t*-tests revealed that relative to partnered mothers, single mothers were more likely to perform the following roles all or mostly on their own: making decisions about their child, *t*(52) = 3.21, *p* = .002, *d* = 0.95, *M*_single_ = 3.35, *SD*_single_ = 0.93, *M*_partnered_ = 2.46 *SD*_partnered_ = 0.96; caring for other family or children at home, *t*(50) = 4.32, *p* < .001, *d* = 0.97, *M*_single_ = 3.41, *SD*_single_ = 0.80, *M*_partnered_ = 2.17, *SD*_partnered_ = 1.04; providing financial support to the family, *t*(52) = 6.42, *p* < .001, *d* = 1.14 *M*_single_ = 3.41, *SD*_single_ = 1.12, *M*_partnered_ = 1.27, *SD*_partnered_ = 1.15; and managing day-to-day chores, *t*(52) = 3.01, *p* = .003, *d* = 1.08, *M*_single_ = 3.35, *SD*_single_ = 1.17, *M*_partnered_ = 2.38, *SD*_partnered_ = 1.04. Among single mothers, all family roles had a mean ≥ 3.29, indicating they did all or most of all roles assessed.

Correlations among variables of interest are displayed in Table 2. For mothers, greater caregiver strain was associated with lower income, *r*(51) = −.31, *p* = .03, and social support, *r*(53) = −.43, *p* = .001, as well as higher general stress, *r*(54) = .61, *p* < .001, and cancer-specific stress, *r*(54) = .54, *p*<.001. Notably, children’s medical characteristics (e.g., treatment intensity, relapse status) were unrelated to variables of interest, so child characteristics were omitted from regressions. As partnership status is likely a confounding variable in our aim to examine the role of family factors in caregiver strain, and was also associated with family role frequency, *r*(54) = −.53, *p* < .001, it was included in regression analyses.

Fathers reported moderate caregiver strain (*M* = 2.59, *SD* = 0.59), low-to-moderate general stress (*M* = 1.75, *SD* = 0.63), and moderate cancer-specific stress (*M* = 2.64, *SD* = 0.64). Fathers also reported moderate-to-high social support (*M* = 3.74, *SD* = 1.04) and high satisfaction with their family roles (*M* = 2.65, *SD* = 0.46). Fathers most frequently reported providing all financial support to the family and sharing equally with their partner on all other roles (Figure 2).

For fathers, greater caregiver strain was associated with lower social support, *r*(30) = −.45, *p* = .01, as well as greater general stress, *r*(30) = .51, *p* = .004, and cancer-specific stress, *r*(30) = .53, *p* = .003. Notably, fathers’ demographic and children’s medical characteristics (e.g., treatment intensity, relapse status) were unrelated to variables of interest (Table 2). Thus, these variables were omitted from regressions. Given that a focal study aim was to evaluate how family factors are associated with caregiver strain, family role satisfaction and frequency were included in regression analyses.

### Exploratory dyadic analyses

Pearson’s correlations explored similarities in mothers’ and fathers’ reports of caregiver strain, family role frequency and satisfaction, social support, cancer-specific stress, and general stress. The number of dyads ranged from 18 to 20, due to missing data. Correlations were significant between mothers’ and fathers’ caregiver strain, *r*(19) = .60, *p* = .01, and cancer-specific stress, *r*(19) = .60, *p* = .01. For correlations between mothers’ and fathers’ family role frequency, positive correlations indicated discordance and negative correlations indicated concordance regarding who completed proportionally more of a role. There was a significant positive correlation, indicating discordance, in reports of communicating with the ill child, *r*(18) = .51, *p* = .03, such that both parents said they did some or all of this role. Significant negative correlations indicated agreement the mother did more: managing the care of their ill child, *r*(19) = − .62, *p* = .01; caring for other family and children at home, *r*(17) = − .69, *p* = .002; and managing household chores, *r*(18) = − .57, *p* = .01. Parents also agreed fathers provided more financial support, *r*(19) = −.68; *p* = .001. There were no dyadic associations between mothers’ and fathers’ family role satisfaction, social support, or general stress. Additionally, paired samples *t*-tests revealed no significant differences between mothers’ and fathers’ reports of caregiver strain, family role satisfaction, social support, general stress, and cancer-specific stress.

### Multivariate models for caregiver strain

A hierarchical regression model examined factors associated with mothers’ caregiver strain. For detailed information on steps 1 and 2, see Table 3. The final overall model was significant and explained 55.2% of the variance in caregiver strain, *F*(7, 42) = 7.41, *p* < .001. Factors associated with greater caregiver strain included greater general stress (*b* = 0.43, *p* = .001), less social support (*b* = − 0.18, *p* = .04), and greater satisfaction with family roles (*b* = 0.23, *p* = .04).

A hierarchical regression model examined factors associated with fathers’ caregiver strain. For detailed information on step 1, see Table 3. The overall model was significant and explained 49.7% of the variance in fathers’ caregiver strain, *F*(5, 22) = 4.34, *p* = .01. The only significant factor was cancer-specific stress, *b* = 0.45, *p* = .02.

## Discussion

This study enhances our knowledge of how the individual and family context influences levels of caregiver strain experienced by both mothers and fathers in the context of advanced pediatric cancer (Brannan and Heflinger 2001; Pai et al. 2007; Jones et al. 2010; Salvador et al. 2015; Edmond et al. 2016; Ozdemir Koyu and Tas Arslan 2021). Across the entire sample, fathers viewed most roles as shared, whereas mothers reported independent roles. Overall, mothers reported more roles relating to the ill child and home, and fathers reported more contributions as a financial provider; this was particularly true when comparing mother–father dyads. In hierarchical regression models, lower social support, higher general stress, and greater satisfaction with family roles were associated with greater strain for mothers, whereas fathers experienced greater caregiver strain in the context of greater cancer-specific stress. Findings underscore the importance of providing support for both mothers and fathers during this stressful period.

Parents experienced moderate strain and general and cancer-specific stress, yet high social support and satisfaction with family roles. Previous research with parents of children with advanced cancer found parents had elevated distress and high social support (Rosenberg et al. 2013), which partially aligns with current findings, though parents in this sample demonstrated slightly greater functioning comparatively. Many parents in our sample were further from initial diagnosis; as research suggests parents experience greater stress and distress initially with declines to normative levels over time, it is possible our sample reflects these adjustments (Dunn et al. 2012; Bakula et al. 2019). A significant bivariate correlation between lower general stress and more time since diagnosis for fathers in our sample supports this idea, though associations between time since diagnosis and stress were not significant for mothers. Interestingly, social support also generally declines over time among families of children with cancer (Hoekstra-Weebers et al. 2001; Wijnberg-Williams et al. 2005), but high levels of social support may reflect a remobilization of support as the disease progresses.

Although mothers and fathers mostly agreed fathers contributed proportionately more to family financial support, and mothers somewhat more to the care of their ill child, there were large discrepancies. Notably, fathers viewed most roles as shared, whereas mothers viewed themselves as the main contributor. Within paired mothers and fathers, there was also disagreement regarding which parent communicated most with the ill child. Although research supports mothers assuming a caregiving role and fathers the role of financial provider (Jones et al. 2010), the number of father-reported shared roles suggests that mothers, healthcare providers, and researchers underestimate fathers’ roles, or that fathers overestimate their roles. Mothers are included more frequently in pediatric cancer research (Pai et al. 2007; Sultan et al. 2016), and fathers have reported feeling disconnected or even unwelcome from pediatric healthcare settings (Jones et al. 2010). Thus, what is known about parental roles during treatment is likely biased by mother perspectives.

For mothers, caregiver strain may be the result of broader family and social contexts. First, mothers’ general and cancer-specific stress were both related to caregiver strain. General stress involves feeling out of control and unable to keep up with life’s demands. Thus, mothers who have pre-existing stressors prior to their child’s diagnosis may have more difficulty managing care. They may also feel less available to other children or feel disconnected from home and work during the illness (Long and Marsland 2011; Long et al. 2018). Consistent with other research (Brannan and Heflinger 2001; Ozdemir Koyu and Tas Arslan 2021; Brennan et al. 2022), mothers with more social support experienced less caregiver strain. Finally, and counterintuitively, greater satisfaction with family roles was associated with greater caregiver strain for mothers. It is possible that mothers were more satisfied when more involved, as was true in associations between frequency and satisfaction with family roles. Thus, although mothers may report caregiver strain, they may appreciate being highly involved. Notably, the lack of association between child medical factors and caregiver strain was unexpected given previous work (Brannan and Heflinger 2001; Salvador et al. 2015; Edmond et al. 2016; Boztepe et al. 2019; Ozdemir Koyu and Tas Arslan 2021). It is possible that advanced cancer and more intensive treatment restricted variability, inhibiting the detection of expected associations (Salvador et al. 2015; Edmond et al. 2016; Boztepe et al. 2019; Ozdemir Koyu and Tas Arslan 2021).

For fathers, greater caregiver strain was related to lower social support and general and cancer-specific stress in bivariate analyses, but only cancer-specific stress remained significant in multivariate models. Thus, fathers may feel more strain in the context of more stressful disease and treatment. Previous literature has suggested fathers may experience informational stress and uncertainty regarding the cause and consequence of the disease (Chesler and Parry 2001; Clarke 2005; Jones et al. 2010). Given fathers were proportionately less involved than mothers in medical care and often served as the financial provider (Clarke 2005; Clarke et al. 2009; Jones et al. 2010; Hjelmstedt et al. 2021), their cancer-specific stress may result from disconnection from their child’s day-to-day care (Jones et al. 2010). Thus, fathers may benefit from more information about the disease and treatment (Chesler and Parry 2001; Clarke 2005; Jones et al. 2010), and more support for managing finances (James et al. 2002). However, although less involved relative to mothers, fathers still reported sharing most family roles. Therefore, additional psychosocial support focused on cancer-specific stress may be warranted.

### Study limitations

Findings should be considered in light of several limitations. First, results are specific to families with advanced cancer. Although advanced cancer research is lacking (Berger et al. 2022), results may not generalize to families of children with a greater prognosis for survival. Second, our sample size limited the ability to control for fathers’ demographic characteristics in regressions and thoroughly explore dyadic associations. Associations in multivariate models may be due to common source variance. Future research should increase both the sample of secondary caregivers and dyads to better understand parent roles and caregiver strain in the context of different family constellations. This would also allow for testing of mixed-method and multi-informant models. Our sample was primarily White and non-Hispanic, which may not reflect the role sharing and burdens experienced in historically underrepresented racial and ethnic groups. Additionally, the family roles questionnaire asked parents the amount they do a task relative to their spouse or partner. Although it was intended that parents consider another caregiver of their child with cancer, single parents may not have known who to consider. Though single parents still reported that they shared some of their family roles, analyses of partnered versus single mothers revealed single mothers did more of 4 out of 7 tasks. Future research should consider more inclusive language in their measures and explicitly identifying care partners with research participants. Lastly, longitudinal data are ideal to examine predictive associations among variables within families and inform interventions.

### Clinical implications

Despite these limitations, this remains one of the first studies to quantitatively examine family roles and factors associated with caregiver strain among mothers and fathers of children with advanced cancer. Medical providers should regularly refer families for interdisciplinary palliative care to help manage symptoms, support communication and advance care planning, and relieve caregiving strain for parents (Wiener et al. 2015). Psychosocial providers should screen for families at-risk for negative outcomes, and address both general and cancer-specific stress, in addition to social support, especially for mothers (Wiener et al. 2015). Social work may also mitigate caregiver strain through provision of financial and insurance-related resources to allow more time, particularly for fathers, to spend with their ill child. Additionally, given mothers and fathers had differing views about roles, healthcare providers might facilitate joint conversations about caregiving and ensure information is shared. Taken together, results suggest caregivers need additional support as they navigate this challenging time.

### Conclusions

In conclusion, this study contributes to limited literature aiming to understand the caregiving experiences of mothers and fathers of children with advanced cancer. To optimally support parent and family well-being, results underscore the importance of considering individual stressors and family context.

## Figures and Tables

**Table 1.** Demographic table

	Mothers *n* = 55		Fathers *n* = 30		Children *n* = 66	
Variable	*M (SD)*	*Min – Max*	*M (SD)*	*Min – Max*	*M (SD)*	*Min – Max*
Age in years	41.33 (6.51)	41.28–40.64	43.93 (6.90)	30.41–57.97	12.12 (4.61)	5–23
Years of education	14.89 (2.75)	10–20	14.80 (2.81)	8–20	–
Years since diagnosis	–		–		2.45 (3.05)	
Number of children in home	2.51 (1.18)	1–6	2.13 (1.33)	1–5	–	
	***n* (%)**					
Partnership status					–	
Partnered						
Married	32 (58.2)		25 (83.3)			
Living with someone	6 (10.9)		1 (3.3)			
Single						
Single	4 (7.3)		1 (3.3)			
Divorced	10 (18.2)		2 (6.7)			
Separated	2 (3.6)		0 (0)			
Widowed	1 (1.8)		1 (3.3)			
Family Income					–	
Under $25,000 per year	13 (23.6)		6 (20.0)			
$25,001–$50,000 per year	10 (18.2)		3 (10.0)			
$50,001–$75,000 per year	7 (12.7)		6 (20.0)			
$75,001–$100,000 per year	7 (12.7)		6 (20.0)			
$100,001–$150,000 per year	6 (10.9)		5 (16.7)			
$150,001 or more per year	9 (16.4)		3 (10.0)			
Missing	3 (5.5)		1 (3.3)			
Relationship to child					–	
Biological parent	50 (90.9)		29 (96.7)			
Stepparent	1 (1.8)		0 (0)			
Grandparent	3 (5.5)		0 (0)			
Aunt/uncle	1 (1.8)		1 (3.3)			
Race					–	
Asian	3 (5.5)		3 (10.0)			
Black or African American	1 (1.8)		1 (3.3)			
White	48 (87.2)		25 (83.3)			
Not listed	3 (5.5)		1 (3.3)			
Ethnicity					–	
Hispanic or Latino	0 (0)		0 (0)			
Not Hispanic	54 (98.2)		30 (100)			
Missing	1 (1.8)		0 (0)			
Child Sex	–		–			
Male					39 (59.1)	
Female					27 (40.9)	
Diagnosis type	–		–			
Leukemia					19 (28.8)	
Lymphoma					5 (7.6)	
Brain tumor					14 (21.2)	
Other solid tumor					28 (42.4)	
Reason for eligibility						
Initial prognosis <60%					22 (33.3)	
Relapsed/refractory disease					44 (66.7)	
Treatment intensity						
Least intensive	–		–		0 (0)	
Moderately intensive					2 (3.0)	
Very intensive					20 (30.3)	
Most intensive					43 (65.2)	
Missing					1 (1.5)	

**Table 2.** Correlations

Measure	1	2	3	4	5	6	7	8	9	10	11	12	13	14	15
1. CSQ	–	−.31	.08	.53**	.51**	−.45*	−.06	.19	−.35	−.08	−.003	−.12	.17	−.03	−.28
2. FRQ satisfaction	.002	–	−.03	−.09	−.52**	.49**	.10	−.002	.43*	.20	.09	.29	.07	.18	.08
3. FRQ frequency	.09	−.32**	–	−.05	−.07	−.45*	−.30	.12	−.03	.04	.21	.14	.28	.06	−.14
4. RSQ-AC	.54**	−.12	.06	–	.56**	−.23	−.28	−.26	−.48**	−.03	.06	−.21	−.06	.10	−.31
5. PSS	.61**	−.23	.03	.52**	–	−.37*	−.30	.01	−.47*	−.08	.05	−.12	−.17	.19	−.37*
6. MOS-SSS	−.43**	.23	.07	−.34**	−.35**	–	.31	−.004	.12	−.05	−.18	−.05	−.35	.25	.06
7. Partnered^†^	−.15	.17	−.53**	−.19	−.04	−.09	–	−.18	.21	−.03	−.22	−.29	.06	−.22	.18
8. Identifies as White race^†^	.16	−.15	.28*	−.04	.17	.13	−.02	–	−.07	−.23	.06	.25	−.01	.06	.08
9. Family income	−.31*	−.06	−.30*	−.22	−.27	.13	.28*	.04	–	.64**	−.08	.17	.10	.02	−.10
10. Education level	−.19	.03	−.25	−.14	−.24	.16	.24	−.06	.65**	–	−.20	.08	−.15	.24	.14
11. Child female sex^†^	−.04	.21	.03	.002	−.08	.29*	.18	.06	−.01	.02	–	−.09	.11	.12	.12
12. Child age	−.05	.29*	.04	−.12	−.17	−.01	−.01	.03	.03	−.03	−.09	–	.02	−.07	.24*
13. Treatment intensity	.24	.22	.06	−.06	−.21	−.10	.01	.02	−.05	−.12	.11	.02	–	−.38**	.25*
14. Initial diagnosis^†^	−.25	.10	−.06	.03	−.05	.26	−.06	−.10	.16	.04	.12	−.07	−.38**	–	−.49*
15. Time since diagnosis	−.05	−.21	.28*	−.01	−.19	−.09	.001	.08	.12	−.08	.12	.24*	.25*	−.49**	–

*Note*: Fathers’ correlations (*n* = 30) are presented above the diagonal; mothers’ correlations (*n* = 55) are presented below the diagonal.

† Refers to dichotomous variables: 1 – partnered versus 0 – single; 1 – White race versus 0 – Other races; 1 – female versus 0 – male; 1 – initial poor diagnosis as reason for study entrance versus 0 – relapsed/recurrent disease.

* *p*<.05;

** *p*<.01

**Table 3.** Hierarchical linear regression results

	*Mothers*						*Fathers*					
Variables	Step	*B* (SE)	*β*	*F*	*R* ^*2*^	*p*	Step	*B* (SE)	*β*	*F*	*R* ^*2*^	*p*
	1			2.58	.10	.09				–	–	–
Constant		3.28 (0.21)				<.001		–				–
Partnered		−0.119 (0.21)	−.08			.57		–	–			–
Family income		−0.103 (0.05)	−.28			.06		–	–			–
	2			10.19	.48	<.001	1			8.06	.39	.002
Constant		1.19 (0.45)				.01		1.06 (0.40)				.01
Partnered		−0.11 (0.16)	−.08			.50		–	–			–
Family income		−0.04 (0.04)	−.10			.41		–	–			–
RSQ-AC mean		0.35 (0.15)	.29			.03		0.40 (0.18)	.43			.03
PSS mean		0.43 (0.12)	.45			.001		0.26 (0.18)	.27			.16
	3			7.41	.55	<.001	2			4.34	.50	.007
Constant		1.26 (0.82)				.13		3.22 (1.15)				.01
Partnered		−0.17 (0.18)	−.12			.34		–	–			–
Family income		−0.006 (0.04)	−.02			.89		–	–			–
RSQ-AC		0.28 (0.15)	.23			.07		0.45 (0.18)	.48			.02
PSS		0.43 (0.12)	.45			.001		−0.02 (0.23)	−.02			.94
MOS-SSS		−0.18 (0.08)	−.25			.04		−0.23 (0.13)	−.39			.08
FRQ satisfaction		0.23 (0.11)	.25			.04		−0.13 (0.26)	−.10			.63
FRQ frequency		0.08 (0.11)	.10			.46		−0.31 (0.22)	−.27			.17
